# Maladie des brides amniotiques: diagnostic anténatal et difficultés de prise en charge (à propos de 02 cas de malformations létales)

**DOI:** 10.11604/pamj.2019.32.116.18048

**Published:** 2019-03-13

**Authors:** Hind Adadi, Hekmat Chaara, Imane Attar, Sofia Jayi, Fatim-Zahra Fdili Alaoui, Moulay Abdelilah Melhouf

**Affiliations:** 1 Service Gynécologie Obstétrique II, CHU Hassan II, Fès, Maroc

**Keywords:** Maladie des brides amniotiques, embryo-fœtopathies, diagnostic anténatal, pronostic, Amniotic band syndrome, embryo-fetopathies, prenatal diagnosis, prognosis

## Abstract

La maladie des brides amniotiques (MBA) est un ensemble de malformations congénitales complexes, intéressant principalement les membres, mais aussi la région cranio-faciale et l'axe thoraco-abdominal. Deux théories principales physiopathologiques s'opposent: la rupture précoce de l'amnios (théorie exogène) conduirait à la formation de brides fibreuses, qui seraient elles-mêmes responsables par strangulation des malformations observées; la théorie endogène privilégie une origine vasculaire, les brides n'ayant alors aucun rôle causal. Le pronostic de la maladie dépend de la gravité des malformations. Afin de discuter des difficultés diagnostiques et thérapeutiques de la maladie des brides amniotiques (MBA), nous rapportons deux cas témoignant de malformations létales de cette maladie. L'objectif principal de ce travail est d'aborder l'intérêt du diagnostic anténatal concernant son influence sur la prise en charge thérapeutique de cette embryo-foetopathie.

## Abstract (en)

Amniotic band syndrome (ABS) comprises of a spectrum of complex congenital malformations mainly interesting the limbs, but even the craniofacial region and the thoracoabdominal axis. There are two major opposing pathophysiological theories: premature rupture of the amniotic sac (exogenous growth theory) would result in the formation of fibrous bands which would lead to strangulation, thus causing the observed abnormalities; the endogenous theory holds that this is a syndrome of vascular origin and that the bands have no causative role. Prognosis depends on the severity of malformations. We here report two cases of lethal malformations in order to discuss the challenges in the diagnosis and treatment of amniotic band syndrome. This study mainly aims to highlight the role of prenatal diagnosis in the therapeutic management of this embryo-fetopathy.

## Introduction

La maladie des brides amniotiques (MBA) est un ensemble de malformations congénitales complexes, intéressant principalement les membres mais aussi la région crânio-faciale, ainsi que l'axe thoraco-abdominal. Ces malformations ont pour caractéristiques d'être asymétriques, polymorphes et de ne respecter aucune systématisation embryologique. Selon le milieu, la MBA peut poser aux praticiens des difficultés de prise en charge non seulement au niveau du plateau technique mais également en ce qui concerne les considérations morales et religieuses. Nous rapportons 02 cas de la MBA, dont le diagnostic fut réalisé en anténatal au sein de notre formation afin de rappeler la diversité des formes cliniques et présenter les difficultés diagnostiques et thérapeutiques dans un contexte de ressources limitées.

## Patient et observation

### Observation N°1

**Figure 1:**
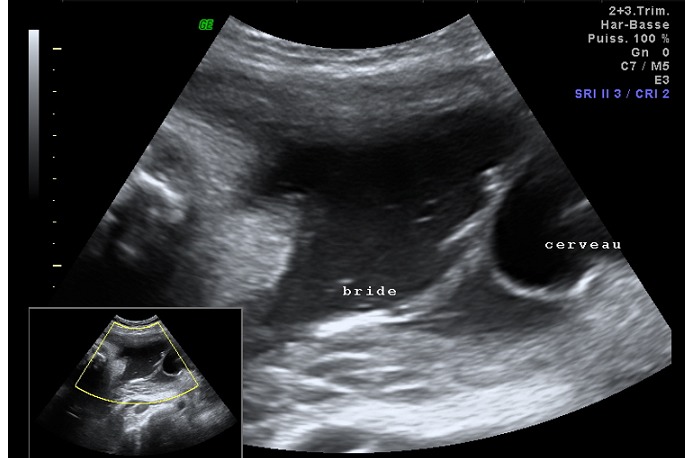
Image échographique d'une bride amniotique entre le défect crânien et la paroi uterine

**Figure 2:**
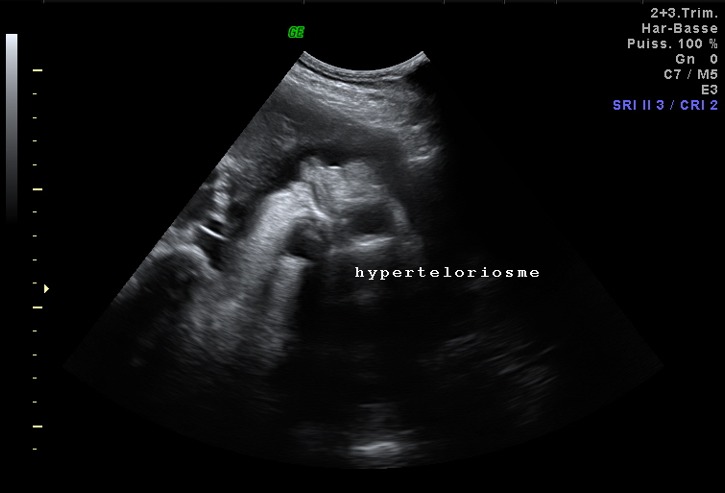
Aspect échographique de la face objectivant un hypertélorisme

Mme A.N âgée de 36 ans, mariage non consanguin, ayant comme antécédents 02 frères diabétiques sous Antidiabétiques Oraux (ADO), primigeste, référée dans notre formation pour prise en charge (PEC) d'une grossesse associée à un diabète gestationnel découvert à 25 semaine d'aménorrhée (SA) par une Hyperglycémie Provoquée Oralement (HGPO) à 75gr de glucose positive à T0 et T1 et dont l'échographie morphologique réalisée au sein de notre formation à 25SA+04jours a objectivé une grossesse monofoetale évolutive, agénésie partielle de la boite crânienne en postérieure, présence du cerveau dans le liquide amniotique sans repères anatomiques des structures avec mise en évidence d'un lien entre le défect crânien et la paroi utérine par des brides amniotiques, l'aspect de la face était difficile à apprécier avec existence d'un probable hypertélorisme. Le reste de la morphologie était sans particularités (Figure 1, Figure 2).

**Figure 3:**
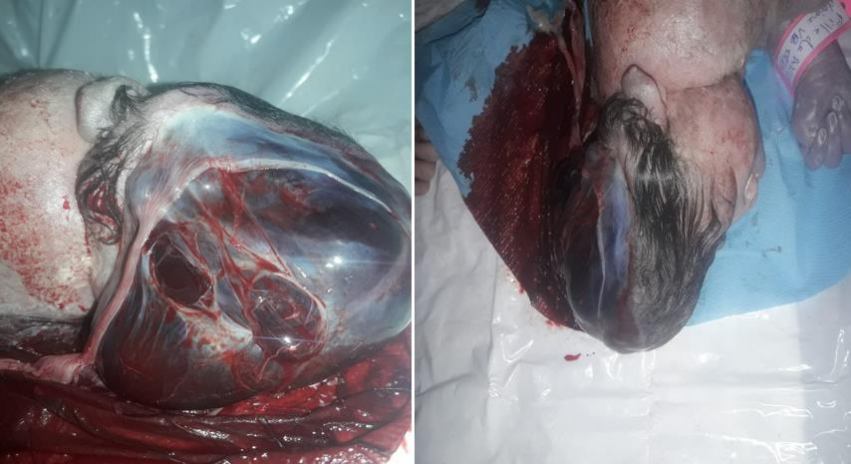
Image du nouveau-né objectivant une agénésie partielle de la voute crânienne avec une adhésion entre le défect crânien et l'amnios couvrant le tissu cerebral

La patiente a bénéficié d'un suivi de grossesse dans notre formation qui s'est déroulé sans incidents. A 37SA+01jour la patiente est rentrée en travail spontanément, donnant naissance suite à un accouchement par voie basse, à un nouveau-né de sexe féminin, poids à la naissance (PDN) à 2000gr, APGAR 02/10 à la 1^ère^ et à la 5^ème^ minute, décédé à 20 minutes de vie. L'examen clinique avait objectivé une agénésie partielle de la voute crânienne avec une adhésion entre le défect crânien et l'amnios couvrant le tissu cérébral (Figure 3).

### Observation N°2

**Figure 4:**
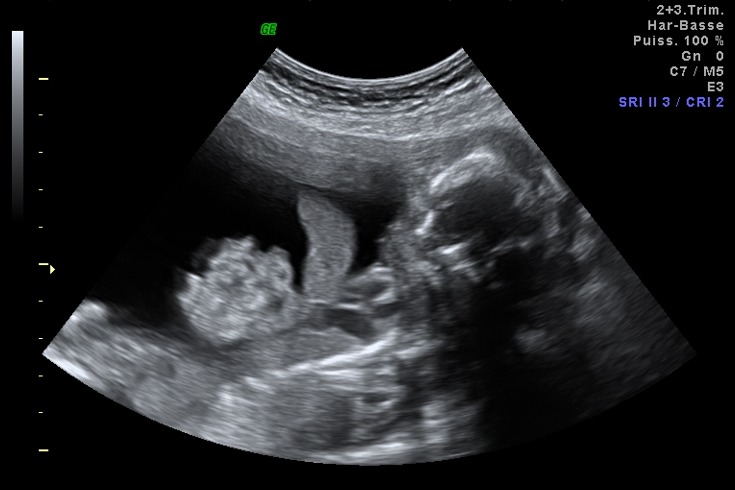
Image échographique objectivant une coelosomie antérieure complete

Mme K.H âgée de 29 ans, mariage non consanguin, sans antécédents personnels ou familiaux notables, G2P1 (1EV/AVB âgé de 03 ans de bon développement psychomoteur), référée dans notre formation par son médecin traitant à 22SA pour suspicion échographique d'une coelosomie antérieure et dont l'échographie morphologique réalisée au sein de notre formation a objectivé une grossesse monofoetale évolutive, coelosomie antérieure complète (cœur, thorax et viscères abdominaux baignant dans le liquide amniotique), cerveau sans particularités mais coincé à gauche et séparé du corps par des brides amniotiques (Figure 4).

**Figure 5:**
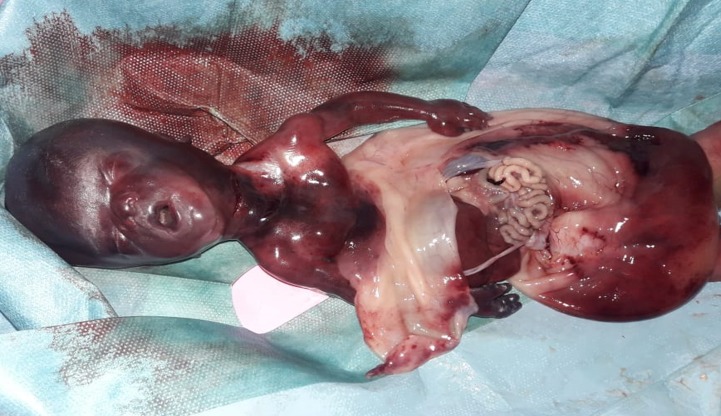
Présence d'un sac amniotique large reliant le rebord cutané du défect pariétal à la surface placentaire et contenant les organes thoraciques et abdominaux

La décision fut de réunir le comité d'éthique pour accord de l'interruption médicale de grossesse (IMG) qui fut jugée recevable vu la létalité de la malformation. Après accord de la patiente et du conjoint, nous avons réalisé un déclenchement par du misoprostol selon le protocole recommandé par la Fédération Internationale de Gynécologie et d'Obstétrique (FIGO) 2017 sous surveillance stricte afin de guetter la survenue d'une éventuelle complication. La patiente a répondu au déclenchement après la 3^ème^ pose donnant naissance par voie basse à un mort-né de sexe masculin, poids à la naissance (PDN) à 600gr. L'examen clinique avait objectivé la présence d'un sac amniotique large reliant le rebord cutané du défect pariétal à la surface placentaire et contenant les organes thoraciques et abdominaux (Figure 5).

## Discussion

La MBA correspond à un ensemble de malformations allant de la constriction et du lymphœdème des doigts aux anomalies congénitales multiples intéressant principalement les membres, mais aussi la région crânio-faciale et l'axe thoraco-abdominal [1, 2]. Ces malformations résultent probablement de multiples processus pathologiques différents [1, 3]. Deux théories ont été discutées pour expliquer sa pathogénie: selon la théorie endogène de Streeter en 1930, il s'agit d'une malformation du disque germinal qui entraîne un dérèglement du développement de l'amnios [4]. La théorie exogène de Torpin en 1965 s'oppose à la précédente; elle est fondée sur la rupture prématurée de l'amnios, dont les débris viennent former des brides constrictives à la surface cutanée fœtale [5]. Malgré de très nombreux travaux, aucune des deux théories n'a pu être démontrée jusqu'à présent. Il est fortement probable que plusieurs mécanismes non exclusifs participent à la formation des différentes lésions de cette maladie. Une anomalie de la vascularisation superficielle du fœtus, en particulier la survenue d'une hémorragie, en serait le principal facteur pathogène [6]. La MBA est une pathologie relativement rare, son incidence est comprise entre 1/1.200 et 1/15.000 naissances vivantes [1, 7]. En Afrique, son incidence n´est pas connue, les cas rapportés proviennent de données hospitalières; beaucoup de cas ne sont certainement pas rapportés. Il convient alors non seulement de renforcer les capacités diagnostiques des praticiens par la formation et l´amélioration du plateau technique mais aussi d´améliorer la notification des cas en mettant en place un registre national des malformations congénitales.

Le diagnostic de la MBA est possible dès le premier trimestre, en fonction de la nature et de la sévérité des malformations observées [8, 9]. Les anomalies crânio-faciales et thoraco-abdominales peuvent être dépistées dès la première échographie à 10-12 SA, alors que les anomalies isolées de membres sont généralement dépistées à l´occasion d´un nouvel examen échographique. Dans notre observation, le diagnostic a été réalisé au 2^ème^ trimestre (respectivement à 22SA et 25SA+04jours). La MBA est caractérisée par l´aspect asymétrique de ses anomalies, ainsi que par l´absence de systématisation embryologique. La détection anténatale d´une bride amniotique n´est pas indispensable à son diagnostic. Cependant, l´examen anatomopathologique devra confirmer le diagnostic [10]. Ainsi, le diagnostic doit être évoqué devant la présence des signes caractéristiques suivants: 1) Constriction ou amputation asymétrique d´une extrémité de membre avec lymphœdème en aval de la constriction; 2) malformations asymétriques crânio-faciales (encéphalocèles, fentes labiopalatines); 3) coelosomies; 4) pseudo-syndactylies; 5) présence d´une bride amniotique au contact du pôle fœtal lésé.

L´absence de vascularisation visible en Doppler couleur et pulsé au sein de ces brides confirme le diagnostic [11]. Dans les pays en voie de développement, comme le Maroc, l'échographie obstétricale n´est pas toujours accessible à toutes les femmes. Dans ces conditions, il est difficile de faire un dépistage précoce des anomalies fœtales et même quand l'échographie est disponible, ce sont des facteurs techniques (faible performance des appareils) et/ou humains (inexpérience de l'échographiste et sa méconnaissance des pathologies malformatives) qui peuvent constituer des limites au diagnostic anténatal. Le pronostic de la MBA lors de la présence de polymalformations cranio-faciales et viscérales, est connu et sombre. La situation est tout autre en cas de constriction superficielle et isolée d´un membre. Concernant nos 02 cas, les malformations rapportées sont sévères, létales et intéressaient d´une part le crâne à type d´encéphalocèle, et d´autre part le thorax et l´abdomen à type de thoraco-abdominoschisis.

Par ailleurs, le pronostic obstétrical des patientes ayant un fœtus présentant une anomalie liée à la MBA n´est pas modifié par rapport à la population générale [2, 12, 13]. En particulier, en cas de malformation cranio-faciale ou viscérale, on ne rencontre pas de taux plus élevé d´anomalies de la présentation ou de dystocie [2, 13, 14]. À la naissance, la quantité de liquide amniotique est considérée comme normale, dans la majorité des cas [2]. Cependant, certains auteurs rapportent un taux majoré de fausses couches tardives et de prématurité en cas de MBA [5, 14]. Dans les 02 cas que nous avons rapportés, les accouchements réalisés par voie basse se sont déroulés sans incidents, la quantité du liquide amniotique était considérée comme normale. Les 02 cas de MBA que nous rapportons étaient au-dessus de toute ressource thérapeutique, le décès est inéluctable soit *in utero*, soit peu après la naissance. L´interruption de la grossesse semble donc raisonnable dans ces cas mais en Afrique, elle est encore difficilement accordée par le comité d'éthique et aussi difficilement acceptée par les patients pour des raisons socioculturelles et religieuses.

## Conclusion

La MBA reste rare, cependant son dépistage doit être systématique en période néonatale. Afin d'améliorer le diagnostic dans les pays en voie de développement, il est nécessaire de créer des centres de référence en génétique humaine et en fœtopathologie, des registres nationaux des malformations congénitales, de disposer d'équipements performants et de spécialistes (échographistes, obstétriciens, pédiatres) très bien avisés sur les malformations de l'enfant. L'attitude obstétricale sera adaptée au cas par cas et dépendra de la gravité du tableau observé. Une interruption médicale de grossesse pourra être proposée lorsque les malformations fœtales seront reconnues comme étant incompatibles avec la vie. Sinon, une prise en charge multidisciplinaire de la grossesse qui reste aussi un challenge dans nos pays à ressources limitées, est indispensable afin d'expliquer au couple la difficulté d'établir en anténatal un pronostic fonctionnel, ainsi que les possibilités thérapeutiques postnatales, voire prénatales.

## Conflits d'intérêts

Les auteurs ne déclarent aucun conflit d'intérêts.
